# Mechanistic insights into Lhr helicase function in DNA repair

**DOI:** 10.1042/BCJ20200379

**Published:** 2020-08-19

**Authors:** Ryan J. Buckley, Kevin Kramm, Christopher D. O. Cooper, Dina Grohmann, Edward L. Bolt

**Affiliations:** 1 School of Life Sciences, University of Nottingham, Nottingham, U.K.; 2 Institute of Microbiology and Archaea Centre, University of Regensburg, 93053 Regensburg, Germany; 3 Department of Biological and Geographical Sciences, School of Applied Sciences, University of Huddersfield, Huddersfield, U.K.

**Keywords:** DNA repair, helicase, Lhr

## Abstract

The DNA helicase *L*arge *h*elicase*-r*elated (Lhr) is present throughout archaea, including in the Asgard and Nanoarchaea, and has homologues in bacteria and eukaryotes. It is thought to function in DNA repair but in a context that is not known. Our data show that archaeal Lhr preferentially targets DNA replication fork structures. In a genetic assay, expression of archaeal Lhr gave a phenotype identical to the replication-coupled DNA repair enzymes Hel308 and RecQ. Purified archaeal Lhr preferentially unwound model forked DNA substrates compared with DNA duplexes, flaps and Holliday junctions, and unwound them with directionality. Single-molecule FRET measurements showed that binding of Lhr to a DNA fork causes ATP-independent distortion and base-pair melting at, or close to, the fork branchpoint. ATP-dependent directional translocation of Lhr resulted in fork DNA unwinding through the ‘parental’ DNA strands. Interaction of Lhr with replication forks *in vivo* and *in vitro* suggests that it contributes to DNA repair at stalled or broken DNA replication.

## Introduction

Lhr (*L*arge *h*elicase*-r*elated) protein is an ATP-dependent DNA translocase and helicase that forms a distinct group within Superfamily 2 helicases [1,2]. Lhr was discovered and named in bacteria [2], in which it is present in eight of ∼30 phyla [2,3]. It is widespread in archaea [4], and the archaeal Lhr is a sequence homologue of the DDX-family of uncharacterized putative helicases found in eukaryotes including in humans [5–7]. Archaeal and bacterial Lhr proteins show high amino acid sequence identity (typically ∼30%) between their N-terminal 800–900 amino acids, which is referred to as the ‘Lhr-Core’, that comprises their helicase domains [8]. Bacterial Lhr is extended to 1300–1500 amino acids by a region of unknown function that lacks obvious sequence homologues. Biochemical analysis of the Lhr-Core from the bacteria *Mycobacterium smegmatis* and *Pseudomonas putida* identified ATP-dependent ssDNA translocation with 3′ to 5′ directionality [1,9,10]. A crystal structure of bacterial Lhr-Core highlights significant similarities with the archaeal DNA repair helicase Hel308 [9,11], most notably in the orientation and interaction of its winged helix domain (WHD) with RecA-like domains typical of Ski2-like helicases [12,13].

Lhr-Core is conserved in many archaea and bacteria, in a genomic context adjacent to a manganese-dependent phosphodiesterase (MPE), an enzyme with active site architecture resembling Mre11 [8]. In other bacteria, full-length Lhr frequently occurs adjacent to the gene encoding RNaseT, which has roles in DNA repair and RNA maturation [14,15]. Deletion of the Lhr-Core gene (Saci_1500) in the archaeon *Sulfolobus acidocaldarius* resulted in a mild, ∼4-fold, sensitivity to UV irradiation in comparison with wild type cells [16]. In contrast, genetic analysis of Lhr in *E. coli* revealed a phenotype in cells treated with the replication inhibitor AZT — deletion of gene *lhr* was synergistic with deletion of the gene encoding the replication-recombination-repair protein RadA [17]. These observations, and reported 4-fold up-regulation in transcription of *lhr* in *M. tuberculosis* in response to mitomycin C [18], suggest that Lhr may be part of a prokaryotic replication-coupled DNA repair pathway. In this work we investigated the properties of Lhr protein from archaea, a homologue of the eukaryotic DDX proteins. We provide evidence that archaeal Lhr interacts with stalled DNA replication, and that the purified Lhr protein has a preference for targeting forked DNA, remodelling it at the fork branchpoint prior to its dissociation.

## Materials and methods

### Molecular cloning of archaeal Lhr

The *lhr* gene (open reading frame mt_1802) from the euryarchaeon *Methanothermobacter thermautotrophicus* (Mth) was first cloned into pBluescript using *Sal*I and *Xba*I restriction endonuclease sites (pEB307) after PCR amplification from Mth genomic DNA (a kind gift from Prof. James Chong, University of York). The Mth *lhr* gene contains an internal *Nde*I restriction site that was altered by silent mutation using QuikChange II site-directed mutagenesis (Agilent). This allowed sub-cloning through a second PCR amplification into pET22b and pT7-7 using *Nde*I and *Eco*RI restriction sites (respectively, pEB352 and pEB353). DNA sequences of these constructs were verified to confirm that plasmids were suitable for protein expression and genetic analysis in *E. coli*.

### Genetic analysis of archaeal Lhr

**Figure 1.**
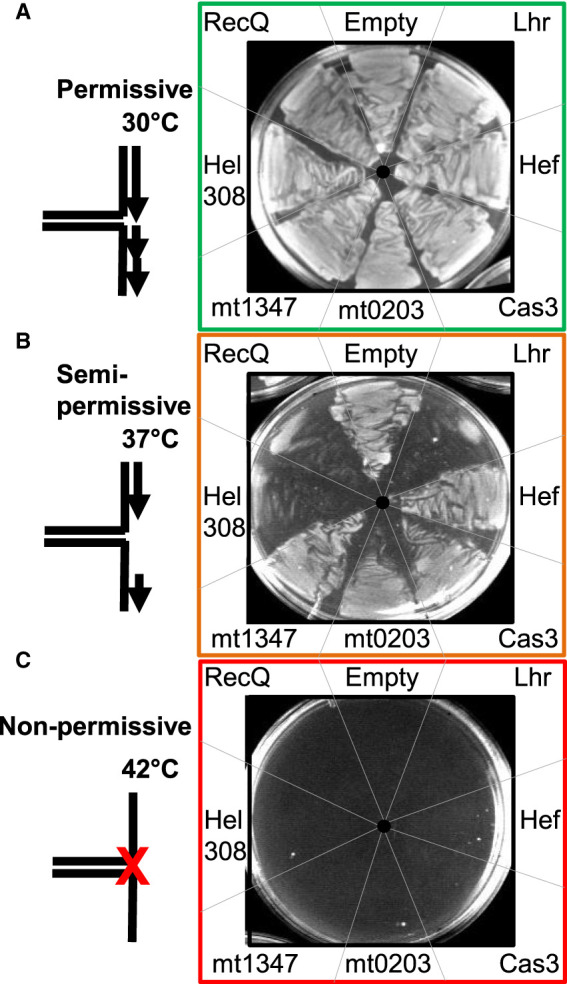
Archaeal Lhr interacts with stalling replication forks in *E. coli dnaE*486 Δ*recQ* cells. Panels are colour-coded to illustrate temperatures at which replication is unhindered (permissive, 30°C), destabilized (semi-permissive, 37°C) or stopped (non-permissive, 42°C). For each temperature cells were spread onto ampicillin agar after expressing the protein indicated from a plasmid. (**A**) At 30°C cells replicate normally resulting in fully viable growth in each sector. (**B**) At 37°C replication is destabilized by the *dnaE*486 allele [20]. Hel308 and RecQ, gave inviability phenotypes as expected [19,20,31], and Lhr gave the same phenotype. (**C**) At 42°C the *dnaE*486 allele makes cells inviable — this is used as a control that *dnaE*486 suppressor mutations have not arisen.

The basis and details for the genetic assay using *E. coli* strain *dnaE*486 Δ*recQ* (Figure 1) are detailed in reference [19]. *E. coli* cells were transformed with empty plasmid vector pT7-7, or with pT7-7 constitutively expressing either bacterial RecQ as a control [20], verified helicases from *M. thermautotrophicus —* Hel308 [19], Cas3 [21] and Hef [22] − or putative archaeal helicases, also from *M. thermautotrophicus* — mt1347 and mt0203. Transformed cells were grown in a shaking water bath at 30°C from colonies inoculated in LB broth containing ampicillin (50 µg/ml), until OD_600_ of 0.5. Then 100 µl of culture was spread onto a sector of each agar ampicillin plate for incubation at 30°C, 37°C or 42°C.

### Purification of archaeal Lhr protein

Plasmid pEB352 was transformed into *E. coli* strain BL21 Codon+ (Agilent) for overexpression of Lhr protein from the archaeon *Methanothermobacter thermautotrophicus*. An overnight culture of this (20 ml) was added to 2 L of LB-ampicillin (50 µg/ml) and chloramphenicol (10 µg/ml) and grown at 30°C with shaking in baffled flasks. At an O.D_600_ of 0.5, Lhr expression was induced by addition of isopropyl-β-d-thiogalactopyranoside (IPTG, 0.8 mM) and growth was continued for a further two hours. Harvested cells were resuspended in buffer C (20 mM Tris.HCl pH 8.0, 10% glycerol, 100 mM potassium chloride and 2 mM DTT) for −80°C storage.

To purify Lhr protein the biomass was thawed on ice, sonicated and clarified by centrifugation. Soluble protein supernatant was loaded in buffer C into a 5 ml HiTrap Heparin column, and Lhr was eluted in a linear gradient of 0.1–1.5 M potassium chloride in buffer C at ∼0.7–0.9 M. Peak Lhr fractions were pooled and loaded directly onto a 16/60 sephacryl S200 column in buffer C, and peak fractions were pooled and dialyzed overnight in buffer C. Dialyzed Lhr was loaded onto a 1 ml HiTrap Q sepharose column and was eluted in a linear gradient of 0.1–1.5 M potassium chloride in buffer C, at ∼0.6–0.8 M potassium chloride. Peak Lhr fractions were pooled and dialyzed into buffer C containing 35% glycerol, and stored as aliquots after flash-freezing for storage at −80°C.

### Preparation of DNA substrates for helicase and DNA binding assays

Nucleotide sequences used to generate all substrates are given in Supplementary Table S1. One DNA strand (900 ng in a 20 µl reaction volume) for each substrate was 5′-end labelled with ^32^P using T4 polynucleotide kinase and γ^32^-P-ATP. The radio-labelled DNA strand was separated from unincorporated γ^32^-P-ATP using a BioSpin 6 column and the resulting labelled DNA was mixed with 900 ng of each appropriate unlabelled strand in 1× SSC buffer (150 mM sodium chloride, 15 mM sodium citrate at pH 7.0), heated to 95°C for 5 min and allowed to anneal by cooling overnight to room temperature. Resulting DNA was mixed with gel loading dye and loaded onto a 10% TBE gel for electrophoresis at 150 volts for 2 h. The gel was then exposed to autoradiography film and the developed film revealed the positions of the desired substrates for excision from the gel. DNA was eluted from excised gel slices by soaking overnight at 4°C in 20 mM Tris.HCl pH 7.5 containing 20 mM sodium chloride. DNA in buffer recovered from gel debris was quantified by scintillation counting using as standards the scintillation counts of samples taken throughout the procedure that were of known DNA mass (ng). This established the final yield of substrate DNA in ng that was converted to a final concentration of DNA (nM) for use in assays.

### Helicase assays and EMSAs

See Supplementary Table S1 for substrates. Helicase reactions were in buffer HB (20 mM Tris.HCl pH 7.5, 2 mM DTT, 100 µg/ml BSA and 7% glycerol) supplemented with 2 mM ATP (at pH 7.5) and 1 mM magnesium chloride. Helicase assays were at 45°C for either 20 min or in reactions over a time course as shown. Reactions were stopped by addition of de-proteinising buffer (1× is 0.625% SDS, 50 mM EDTA and 2.5 mg/ml proteinase K) and gel loading dye was added prior to electrophoresis at 150 volts for 1 h through a 10% acrylamide TBE gel. Assay products were imaged on a storm™ scanner (Amersham) from phosphorimaging screens, after drying the gels under a vacuum on a flatbed gel dryer. Assay products were quantified from TIF files of gel images using the GelEval software. For EMSAs, Lhr (100 nM) was mixed with DNA (10 nM) in buffer HB at room temperature with reactions loaded directly onto a 5% acrylamide TBE gel and were imaged using the ChemiDoc MP imaging system (Bio-Rad).

### Assays using fluorescent DNA fork-2 and confocal single-molecule FRET measurements

Fluorescent fork-2 DNA was formed from the four fork-1 oligonucleotides (Supplementary Table S1) mixed in equimolar concentration (10 µM) in annealing buffer (10 mM Tris.HCl pH 7.8, 50 mM NaCl, 1 mM EDTA), heated to 95°C for 3 min and cooled to room temperature (23°C) over 1.5 h. DNA was stored at −20°C. For EMSAs, Lhr (100 nM) was mixed with DNA (10 nM) in buffer HB at room temperature with addition of ATP and magnesium chloride (1 : 2 mM) as indicated in Figure 3, and reactions loaded directly onto a 5% acrylamide TBE gel. Gels were imaged using the ChemiDoc MP imaging system (Bio-Rad).

Prior to FRET measurements, the sample chambers (Cellview slide, Greiner Bio-One) were passivated with 2 mg/ml BSA in 10 mM Tris.HCl pH 8.0 for 10 min and washed once with Millipore water. For formation of complexes, 1 nM DNA, 1 µM LHR, 1 mM MgCl_2_ and 2 mM ATP were mixed in H78 buffer (20 mM NaHEPES pH 7.8, 10% (v/v) glycerol, 100 mM potassium acetate, 1 mM EDTA, 2 mM DTT) and incubated for up to 20 min at room temperature or 45°C. Afterwards, samples were diluted by a factor of 10 in H78 buffer and added to the sample chamber.

Single-molecule fluorescence of diffusing complexes was detected with a MicroTime 200 confocal microscope (PicoQuant) equipped with pulsed laser diodes (532 nm: LDH-P-FA-530B; 636 nm: LDH-D-C-640; PicoQuant/cleanup filter: zet635; Chroma). The fluorophores were excited at 20 µW using pulsed interleaved excitation (40 MHz). Emitted fluorescence was collected using a 1.2 NA, ×60 microscope objective (UplanSApo ×60/1.20W; Olympus) and focused through a 50 μm confocal pinhole. A dichroic mirror (T635lpxr; Chroma) was used to separate donor and acceptor fluorescence. Additional bandpass filters (donor: ff01-582/64; Chroma; acceptor: H690/70; Chroma) completed spectral separation of the sample fluorescence. Each filtered photon stream was detected by an individual APD (SPCM-AQRH-14-TR, Excelitas Technologies) and recorded by a time-correlated single photon counting (TCSPC) capable HydraHarp 400 (PicoQuant).

### FRET data analysis

Data analysis of confocal FRET measurements was performed with the software package PAM [23]. Photon bursts of diffusing molecules were selected based on an all-photon burst search (APBS, parameters: L = 100, M = 10, and T = 500 μs) and an additional dual-channel burst search (DCBS, parameters: L = 100, M_GG+GR_ = 20, M_RR_ = 20, and T = 500 μs).

For an APBS, the FRET efficiency of each burst (calculated as proximity ratio *E*_PR_) and the raw stoichiometry factor *S*_raw_ was calculated as:

1 $$E_{\mathrm{PR}}=\frac{N_{\mathrm{DA}}}{N_{\mathrm{DD}}+N_{\mathrm{DA}}}$$

2 $$S_{\mathrm{raw}}=\frac{N_{\mathrm{DD}}+N_{\mathrm{DA}}}{N_{\mathrm{DD}}+N_{\mathrm{DA}}+N_{\mathrm{AA}}}$$

where *N*_DD_, *N*_DA_ and *N*_AA_ are the number of detected photons. Indices refer to donor donor emission upon donor excitation (DD), acceptor emission upon donor excitation (DA) and acceptor emission upon acceptor excitation (AA). These were used to calculate the donor leakage and direct excitation correction factors. For DCBS, the FRET efficiency *E* and the stoichiometry factor *S* of each burst were calculated as:

3 $$E=\frac{N_{\mathrm{DA}}-(c_{\mathrm{leak}}\cdot N_{\mathrm{DD}}+c_{\mathrm{dir}}\cdot N_{\mathrm{AA}})}{\gamma \cdot N_{\mathrm{DD}}+N_{\mathrm{DA}}-(c_{\mathrm{leak}}\cdot N_{\mathrm{DD}}+c_{\mathrm{dir}}\cdot N_{\mathrm{AA}})}$$

4 $$S=\frac{\gamma \cdot N_{\mathrm{DD}}+N_{\mathrm{DA}}-(c_{\mathrm{leak}}\cdot N_{\mathrm{DD}}+c_{\mathrm{dir}}\cdot N_{\mathrm{AA}})}{\gamma \cdot N_{\mathrm{DD}}+N_{\mathrm{DA}}+\beta \cdot N_{\mathrm{AA}}-(c_{\mathrm{leak}}\cdot N_{\mathrm{DD}}+c_{\mathrm{dir}}\cdot N_{\mathrm{AA}})}$$

where *c*_leak_ is the correction factor for donor leakage, *c*_dir_ is the correction factor for direct excitation of the acceptor, *γ* and β are the detection and excitation correction factors. Burst data were corrected for donor leakage and direct excitation of the acceptor (determined from APBS according to [24], as well as γ and β (determined from DCBS ES-histograms using an internal fit on multiple E/S separated FRET populations). The data were binned (bin size = 0.025), plotted as E histogram and fitted with a single (DNA) or multiple Gaussian fits using the Origin software.

The inter-fluorophore distance *r* was calculated from corrected *E* values according to:

5 $$r=R_{0}\sqrt[{6}]{\frac{1-E}{E}}$$

using the following Förster radius: *R*_0_ = 5.9 nm of the ATTO 532-ATTO 647N dye pair.

### Analysis of Lhr and DDX52 structures

Protein sequence homology was assessed using BLASTP [25] against sequences with a Protein DataBank [26] record, using the *Methanothermobacter thermautotrophicus* ΔH open reading frame Mth1802 (UniProt: O27830) and human DDX52 (UniProt: Q9Y2R4) helicase protein sequences as search queries. Protein fold, secondary structure and structural homology searches were performed with Phyre2 [27] under Intensive mode. Predicted structure models were analyzed, superimposed and RMSD calculated with DALI [28], superimposing against the *M. smegmatis* Lhr [9] (PDB: 5V9X) helicase structure. Protein secondary structure was predicted in PSIPRED [29]. Structural models rendered in PyMOL were superimposed using the C_α_ chain.

## Results

### Genetic analysis of archaeal Lhr indicates interaction with stalled DNA replication

Lhr is distributed throughout the archaeal domain, including in all classes of the Asgardarchaeota that is most closely related to eukaryotes, and in the extremely reduced genomes of Nanoarchaeota — details are presented as Supplementary Data in Supplementary Table S2. We utilized Lhr from the euryarchaeal species *Methanothermobacter thermautotrophicus* (Mth), and first analyzed this Lhr using genetics. Two previous studies in archaea had deleted the *lhr* gene — in *Haloferax volcanii* this gave no discernible phenotype in response to UV or γ irradiation [30], and in *Sulfolobus acidocaldarius* there was very modest (4-fold) UV sensitivity [16]. Here, we observed a robust phenotype from Mth Lhr in a genetic assay that detects interaction with stalled DNA replication [20]. This assay uses *E. coli* cells with a conditional mutation in the gene encoding DNA polymerase III (*dnaE*), the replicative polymerase. This particular mutation, *dnaE*486, causes structural instability of DNA polymerase III at 37°C that triggers stalling of DNA replication, mimicking DNA damage. Cells survive this by activating replication-coupled DNA repair, therefore 37°C is called a ‘semi-permissive’ temperature. However, interference with de-stabilised replication at 37°C by heterologously expressed protein causes low cell viability because native replication-coupled repair is impeded. This assay had previously identified DNA repair phenotypes for archaeal Hel308 and RecQ [19,20,31], and was re-visited to assess other putative archaeal helicases including Lhr (Figure 1). As expected from previous findings [19,20], expression of bacterial RecQ or Hel308 in these cells at permissive temperature (30°C) had no effect on viability (Figure 1A), indicating that these proteins are not toxic when expressed in *E. coli* cells replicating normally, but both caused inviability at 37°C indicating interaction with unstable replication (Figure 1B). Expression of Lhr also caused cell inviability at 37°C, and the normal viability of cells at 30°C confirmed that Lhr protein does not confer toxicity to normal replication. Expression of other known or putative archaeal helicases had no observable effect on cell viability at 37°C (Figure 1A,B). All cells were inviable at 42°C (Figure 1C), a temperature at which the replisome cannot function because of the *dnaE*486 mutation — this ensures that suppressor mutations have not arisen to give false positive results at 37°C. This genetic analysis suggests that Lhr, like archaeal Hel308 and bacterial RecQ, interacts with de-stabilised replication forks. This information was taken forward for biochemical analysis of the Mth Lhr protein.

### Archaeal Lhr protein preferentially targets fork-DNA for DNA translocation

**Figure 2.**
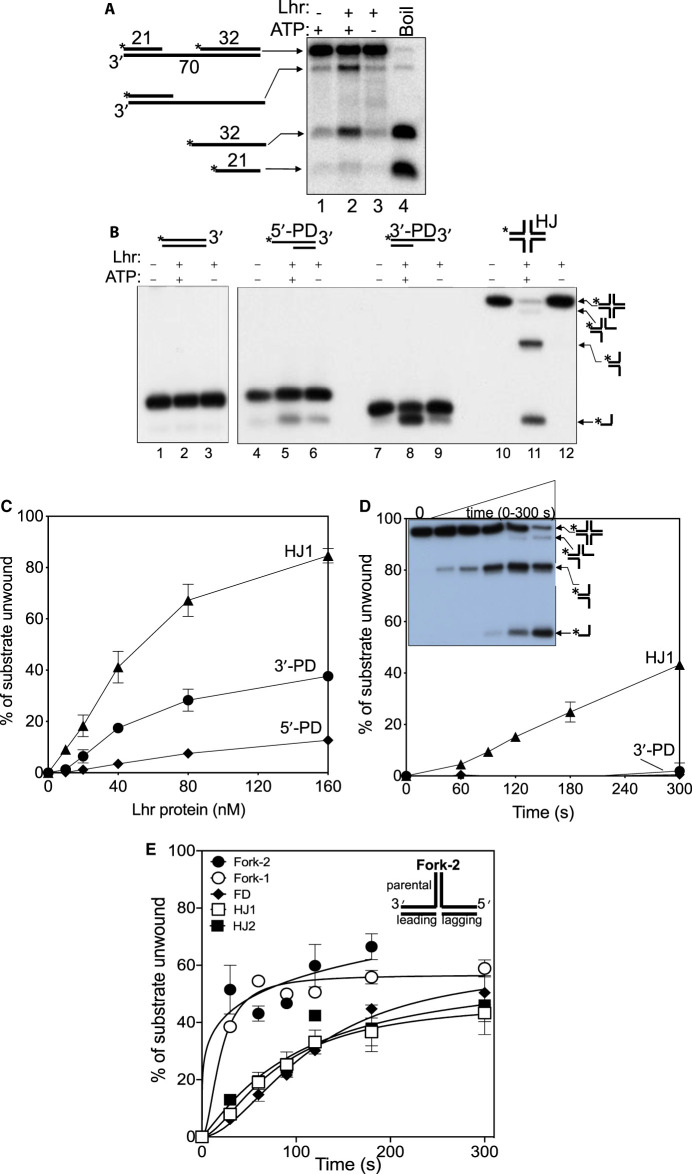
Lhr is most effective at unwinding branched DNA molecules. All parts show results of Lhr helicase reactions observed in TBE 10% acrylamide gels. Asterisks indicate 5′-^32^P end labelling of a DNA strand and DNA was used at 10 nM unless stated. (**A**) Lhr (100 nM) gave ATP-dependent displacement of the 32 nt strand from the gapped duplex (1 nM) indicating 3′ to 5′ directionality. (**B**) Lhr (100 nM) did not significantly unwind fully base paired DNA duplex or a partial duplex with a 5′ ssDNA tail (5′-PD, lanes 4–6), but unwound a partial duplex DNA with a 3′-ssDNA-tail (3′-PD, lanes 7–9). A Holliday junction (HJ) was unwound more effectively in this assay to generate three-strand, two strand and ssDNA products as indicated at the side of the gel panel. The apparent proficiency of Lhr in unwinding the Holliday junction compared with partial duplex DNA Holliday junction was confirmed in part (**C**), in which Lhr was added to DNA at 10, 20, 80 and 160 nM as indicated. Reactions were repeated three times — the range of standard error is shown. (**D**) Holliday junction DNA (HJ) was unwound by Lhr (40 nM) at least 10-fold more effectively than the 3′-tailed partial duplex (3′-PD) over the course of time (0–300 s). The inset gel summarizes that Lhr unwound Holliday junctions into products that were further unwound, indicating that Lhr is not specific for targeting Holliday junctions. Reactions were carried out twice, and bars show the standard error. (**E**) Lhr (40 nM) unwound fork-1 and fork-2 DNA most effectively over time, compared with Holliday junctions. A cartoon of the fork-2 structure is shown for reference to the fork parental, leading and lagging strands. For comparison, the graph also shows a flayed duplex (FD) DNA, comprising the fork parental duplex but neither lagging nor leading strands. Details of the substrates are given in the Supplementary data along with representative gels. Reactions were done three times and bars show standard error from mean.

The bacterial ‘core’ Lhr (Lhr-Core), which lacks a 700 amino acid C-terminal region present in the bacterial but not archaeal Lhr enzymes, is a ssDNA-stimulated ATPase that translocates ssDNA with 3′ to 5′ directionality [10]. Purified full-length archaeal Lhr (Supplementary Figure S1) was challenged with a gapped DNA duplex substrate to determine if it had similar properties (Figure 2A). In this assay, loading of Lhr onto ssDNA revealed 3′ to 5′ translocation directionality by displacement of the 32 nt strand in preference to the 21 nt strand (Figure 2B lanes 2 and 3). DNA unwinding of the gapped duplex by Lhr *in vitro* was most effective at 2 mM ATP and 1 mM magnesium chloride (Supplementary Figure S2), conditions that were used for subsequent assays. We next assessed unwinding of different model synthetic DNA substrates to establish if Lhr had a substrate preference that could be used to gain insight into its DNA unwinding mechanism. In agreement with a requirement for ssDNA to trigger DNA translocation, Lhr did not unwind DNA in a fully base-paired DNA duplex (Figure 2B lanes 1–3). It was weakly active at unwinding a partial duplex with 25 nt of 5′ tailed ssDNA (5′-PD, lanes 4–6) but substantially unwound a partial duplex with a 3′ ssDNA tail (3′-PD, lanes 7–9). This is in agreement with the 3′ to 5′ directionality observed when unwinding the gapped duplex (Figure 2A), but some dissociation of the 5′ tailed substrate suggested that Lhr may more generally distort DNA base-pairing, leading to low levels DNA strand dissociation, when bound to DNA – further investigation of this is presented later (Figure 4). Lhr also unwound a partial duplex comprising an RNA–DNA hybrid with a 3′ single stranded ‘tail’ as well as the corresponding tailed DNA duplex (Supplementary Figure S3). These data indicate that Lhr requires single-stranded DNA (ssDNA) to trigger directional translocation/helicase activity.

**Figure 3.**
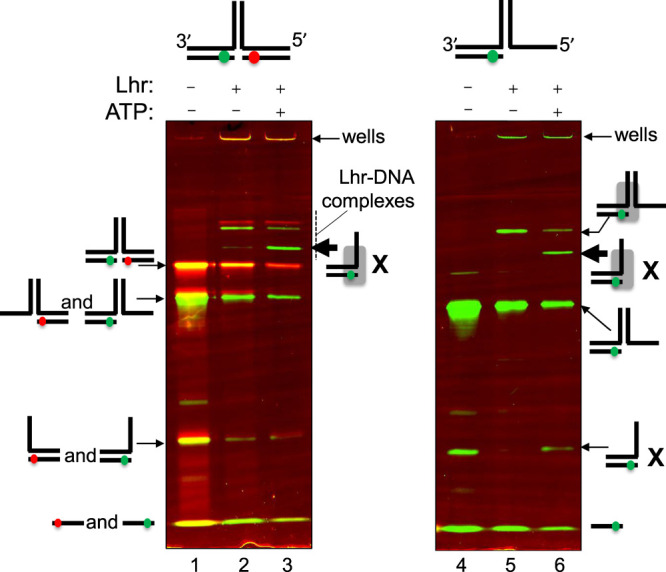
Evidence for directionality of fork dissociation by Lhr. Both panels are from the same native acrylamide EMSA gel, divided to be able to annotate each part with substrate and product DNA. Fork-2 was labelled with ATTO 532 (green) and ATTO 647N (red) fluorophores at the indicated positions in the cartoon representations shown above the gels, a full fork corresponding to lanes 1–3, and a partial fork lacking a lagging strand corresponding to lanes 4–6. The fluorescence signal of the fluorophores was detected using a fluorescence scanner. Green and red bands in the gel correspond to fork-2 and fork-2-Lhr bound reaction products that contain one or both of the labelled DNA strands. Lanes 1 and 4 show bands corresponding to each full substrate as naked DNA, and its component intermediate DNA molecules; each form as shown to the side of the panel. Addition of Lhr (100 nM) and ATP-Mg^2+^ to reactions is indicated above each panel. Free substrate (lanes 1 and 4) was bound by Lhr (lanes 2 and 5), and indicated by the label for Lhr-DNA complexes and a grey rectangle denoting DNA-bound protein. Addition of ATP triggered fork dissociation into the products indicated with the letter X. Helicase products could remain bound to Lhr protein, as indicted by the grey rectangles representing Lhr. ATP-dependent formation of Lhr-bound two-strand DNA product is highlighted in lanes 3 and 6.

**Figure 4.**
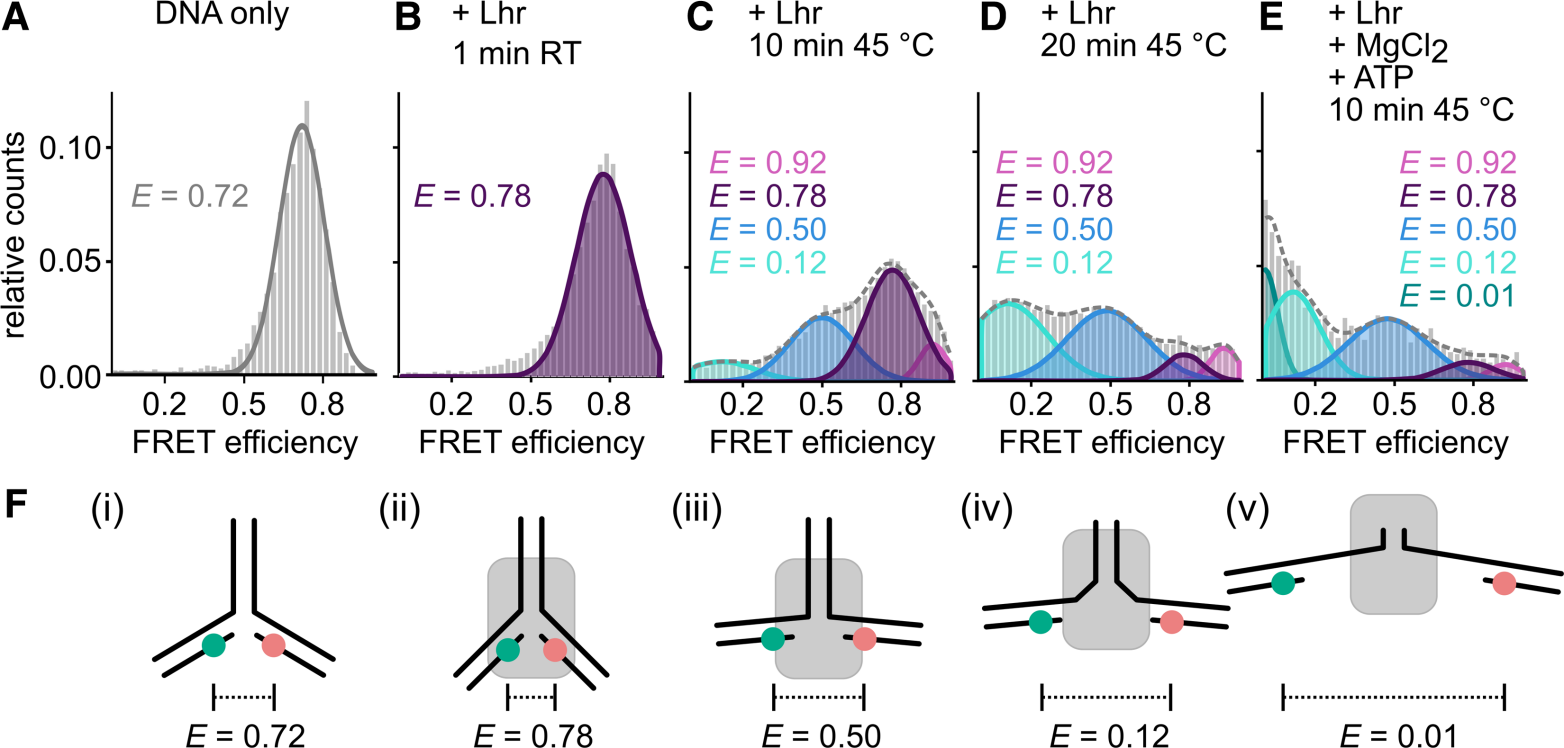
Single-molecule FRET analysis of conformational changes induced by Lhr on fork-2 DNA. Measurements were performed on freely diffusing DNA/protein complexes to monitor Lhr-induced conformational changes on forked DNA substrate. (**A**) ATTO532 (donor) and ATTO647N (acceptor) labelled fork-2 in the absence of protein, (**B**) after addition of Lhr (1 µM) at room temperature (RT), then (**C** and **D**) after 10 min and 20 min of incubation at 45°C. (**E**) After 10 min at 45°C with addition of 1 mM MgCl_2_ and 2 mM ATP. FRET populations were fitted with multiple Gaussian distributions. The mean FRET efficiency *E* of the fitted peaks are shown. The dashed line is the cumulative fit curve. Each measurement was carried out at least three times — see Supplementary Figure S6). (**F**) Putative model for the mechanism of Lhr-dependent fork DNA unwinding. Conformations are based on inter-fluorophore distances derived from the measured FRET efficiencies that are presented as a data table in Supplementary Figure S6: i. relaxed conformation of fork DNA labelled with donor (green, leading strand)) and acceptor (red, lagging strand); ii. compacted fork bound by Lhr (grey); iii. stretched conformation after heat activation of LHR; iv. partially melted fork DNA after ATP-Mg^2+^ addition; v. mostly unwound fork still bound to Lhr.

Unwinding of the 3′ tailed partial duplex (3'-PD) was quite modest — maximally 30% of substrate was unwound when Lhr was used at 10-fold molar excess over DNA (Figure 2C). Lhr unwound an equivalent branched substrate, a Holliday junction (indicated as HJ in the figures), 3-fold to 10-fold more effectively than tailed duplexes measured in, respectively, endpoint (Figure 2C) and time course assays (Figure 2D). This Holliday junction (HJ1) was generated by annealing of the same DNA strand, and its complements, that was used to generate the 3′ tailed partial duplex to ensure DNA sequences were consistent, as detailed in Supplementary Table S1. Lhr generated two major products from unwinding of HJ1 — these products were identifiable as labelled in Figure 2D by comparing them with the single forked product generated by the Holliday junction specific helicase RuvAB (Supplementary Figure S4A), and with ssDNA product of Lhr unwinding the 3′ tailed duplex (Figure 2B lanes 8 and 11, and Supplementary Figure S4B). The structural specificity of RuvAB for unwinding Holliday junctions to only a fork without further unwinding of the fork into ssDNA [32,33], therefore contrasted with Lhr, suggesting that Lhr may be able to target forked DNA for unwinding.

To narrow down the substrate preferences of Lhr *in vitro* we compared unwinding of forked DNA with Holliday junction DNA as a function of time (Figure 2E). Two different Holliday junctions were compared with equivalent forked DNA that comprised a fully base-paired ‘parental’ DNA duplex and leading and lagging strand duplexes of the same DNA sequences as Holliday junctions (Figure 2E and Supplementary Table S1). These assays, using 20 nM of DNA and 40 nM of Lhr protein, indicated modest preference for forked DNA compared with Holliday junctions (Figure 2E). Multiple products from Lhr unwinding Holliday junctions were again apparent (Supplementary Figure S5).

The preference of Lhr for forked DNA that we observed *in vitro* is consistent with Lhr targeting replication forks in genetic assays (Figure 1). But it raised the question, how does Lhr most effectively unwind fully base-paired forks, when it requires access to ssDNA for translocation leading to DNA unwinding? We reasoned targeting of a fork branch-point by Lhr may disrupt base pairing allowing ssDNA loading and translocation, which we investigated using single-molecule Förster resonance energy transfer (smFRET) measurements.

### smFRET measurements reveal ATP-independent remodeling of a DNA fork by Lhr, and ATP-dependent dissociation of the fork-lagging strand

Lhr unwound model DNA forks most effectively in ensemble reactions *in vitro* (Figure 2E), therefore the same fork-2 substrate was used for smFRET analysis. Here, a donor-acceptor dye-pair was positioned in the fork lagging strand (ATTO 647N) and leading strand (ATTO 532) (Supplementary Table S1). We began by assessing Lhr binding and unwinding of this fork-2 in EMSAs, exploiting the dual ATTO labelling that allows for greater differentiation of reaction products than the single ^32^P end-radiolabel (Figure 3 lanes 1–3). The reactions were not de-proteinised and consequently LHR in complex with either the complete fork substrate or unwinding intermediates was detected. In reactions lacking ATP, Lhr-fork DNA complexes were observed (Figure 3 lane 2). With ATP, Lhr helicase products primarily result from unwinding of the fork ‘parental’ DNA not fork lagging or leading strands, visible as a single product. The resulting green fluorescing DNA-LHR complex is consistent with the two-strand molecule indicated that would be generated by 3′ to 5′ directionality of Lhr (product X in lane 3). To verify this, we repeated the Lhr binding and unwinding reactions using a partial fork-2 that lacked the red fluorescing lagging strand (Figure 3 lanes 4–6). As expected in the absence of ATP, Lhr bound to the partial fork-2 resulting in a single complex representing Lhr-DNA binding (lane 5). Addition of ATP gave the same two-strand DNA product both bound with Lhr and not bound (both marked X in Figure 3 lanes 4–6), also consistent with the green ATTO labelled partial fork-2 being unwound 3′ to 5′ through the ‘parental’ duplex.

Having gained some qualitative insight into unwinding of the ATTO labelled fork-2 by Lhr we next assessed the effect of Lhr on DNA conformation within the fork at the single-molecule level (Figure 4), by determining the efficiency of energy transfer from donor to acceptor. Higher FRET efficiency (E) values denote shorter inter-dye distances giving a readout of fork conformation at the branchpoint. In the absence of Lhr, the fork DNA gave a single population (E = 0.72) (Figure 4A,Fi) representing a relaxed state with angles of ∼130° between the lagging and leading strand DNA. Addition of Lhr at room temperature in buffer without ATP-Mg^2+^ shifted the signal to E = 0.78, representing a shortening of the inter-dye distance due to fork compaction or DNA rotation induced by Lhr (Figures 4B and 3F ii). Activating Lhr at 45°C (but without ATP-Mg^2+^) resulted in significant additional FRET populations corresponding to fork DNA undergoing changes into both stretched (E = 0.50) and further compacted (E = 0.92) conformations (Figure 4C,F iii). In these conditions, we also observed decreased signal intensities for compacted forks (E = 0.92 and E = 0.78) that corresponded with an increase in the low FRET efficiency population (E = 0.12), representing a highly stretched or partially unwound fork DNA conformation (Figure 4D,F iv) — the increased inter-dye distance indicated disruption of multiple base pairs close to the fork branch-point. The data indicate that fork DNA binding by Lhr in the absence of ATP causes multiple changes in fork conformation, including partial melting of DNA close to the fork branch-point. Addition of ATP-Mg^2+^ resulted in disappearance of the stretched fork signal (E = 0.5, Figure 4E) and appearance of a population with E ∼ 0 that results from the fork being mostly unwound, fully separating the FRET dye pair (Figure 4E,F part v).

## Discussion

Lhr protein is highly conserved throughout archaea and has sequence homology with DDX damage repair proteins found in humans and other eukaryotes [7]. Lhr proteins form two sub-groups, Lhr and Lhr-Core, the latter including the archaeal proteins of 800–900 amino acids arranged into RecA-like and accessory domains required for helicase activity. Bacterial *lhr* and bacterial/archaeal *lhr-core* are often located in a conserved genome context with at least one gene encoding a nuclease enzyme; *lhr* with *rnt* that encodes a 3′ to 5′ exonuclease implicated in DNA repair [14,15], and *lhr-core* with MPE, a manganese dependent exonuclease [10]. Our observation of a replication phenotype from expression of archaeal Lhr (Figure 1) is consistent with a role in replication-coupled DNA repair suggested from genetic analyses of Lhr from *E. coli* and *M. tuberculosis* [17,18]. It is also consistent with our data from *in vitro* helicase assays (Figures 2 and 3) and smFRET (Figure 4) showing that purified Lhr protein targets and unwinds DNA forks.

The 3′ to 5′ directional DNA translocation of archaeal Lhr is the same as bacterial Lhr [10], and in addition we observe a strong preference for unwinding of DNA within three- or four-stranded forked and Holliday junction molecules, compared with ssDNA tailed-duplexes. Mycobacterial Lhr-Core was most active on RNA–DNA hybrids that have a 3′ ssDNA tail, although 3- or 4-strand forks or Holliday junctions were not tested [1]. Lhr does not seem to be a bona fide Holliday junction ‘branch migration’ helicase because it unwound model forked DNA better than model Holliday junctions, and because the products formed by Lhr unwinding Holliday junctions differed from the RuvAB branch migration complex. In addition, previous genetic studies on bacterial Lhr showed no strong phenotypes for Lhr associated with RuvABC or RecG-promoted recombination-repair, either epistatic or synergistic.

**Figure 5.**
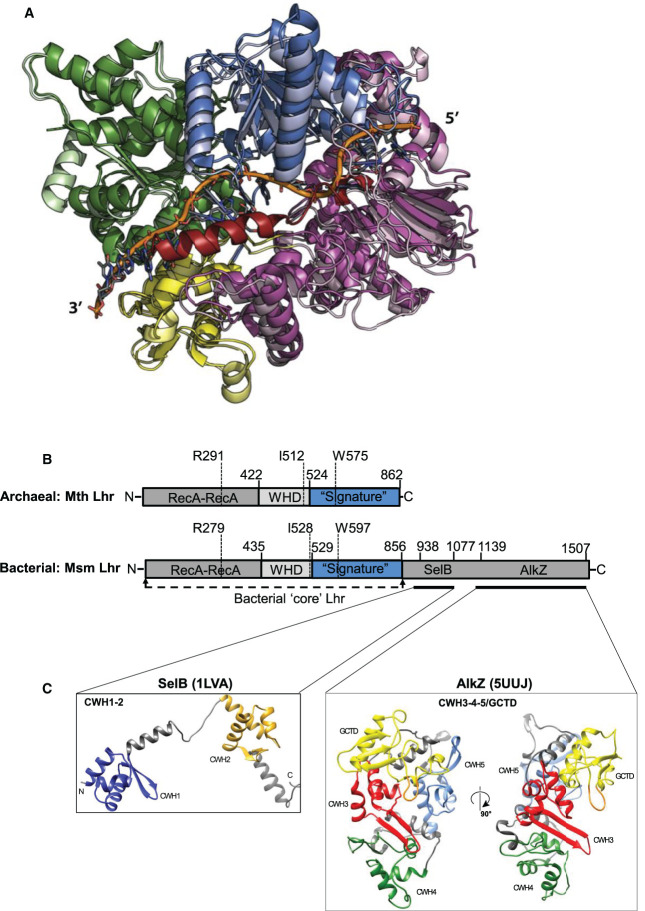
Novel features of Lhr protein structures. (**A**) Structural model of the archaeal Lhr used in this work (Mth1802) superimposed onto *M. smegmatis* Lhr (PDB: 5V9X). Lhr is orientated and coloured according to the original description [9] (green, RecA domain 1; blue, RecA domain 2; yellow, winged helix; pink, domain of unknown function), with the Mth_1802 model in lighter shades. The ssDNA in the Lhr structure is shaded orange, and the *ab initio* modelled Mth1802 C-terminal 30 residues referred to in the text is shaded red. (**B**) Cartoon summary of the domain organization of Lhr proteins from archaea and bacteria. Labelled are the tandem RecA-like domains, winged helix domains (WHD) and a ‘signature’ domain of Lhr proteins that is of unknown functions. Amino acid positions are indicated, including invariant amino acids that are required for helicase activity of the bacterial Lhr [9]. Also highlighted is the ‘core’ helicase of the bacterial Lhr protein, and the C-terminal region of bacterial Lhr that is absent in archaea. (**C**) Summarizes two parts of the bacterial C-terminal Lhr region that match with structural folds of AlkZ and SelB proteins: CWH, C-terminal winged helix-turn-helix motif; GCTD, glycosylase C-terminal domain.

Our data showed more efficient unwinding of DNA forks by Lhr compared with unwinding of DNA from 3′ ssDNA tail provided to load Lhr for 3′ to 5′ translocation. This was despite the forked substrates being fully base-paired. Using single-molecule FRET we observed substantial melting and remodeling of the fork-2 substrate that would yield the ssDNA needed to trigger the ATP-dependent DNA translocation, thus unwinding the fork. The crystal structure of a mycobacterial Lhr-Core helicase bound to ssDNA most closely resembles the DNA repair helicase Hel308 [9,11,34], another Ski2-like helicase which has the same genetic phenotype as Lhr reported in this work and in previous studies [19,31]. The Lhr crystal structure represents the active translocation stage of Lhr, and the archaeal Lhr used in this work superimposes well when structurally modelled against it (RMSD 0.8 Å). including a region of the core bacterial and archaeal Lhr proteins, approximately amino acids 520–860, that is of unknown function that has been referred to as a ‘signature’ domain for Lhr proteins ([9] and Figure 5A). In addition, PHYRE2 *ab initio* modelling and PSIPRED searches of archaeal Lhr both predicted additional alpha helical content that was not resolved in the mycobacterial structure, including a 30-residue alpha helical extension intriguingly positioned relative to RecA-like domains and the translocating DNA strand (Figure 5A). We speculate that this may be significant for additional Lhr-DNA interactions, including with forked DNA, although it has not been possible to model a forked DNA structure onto these structures. Lhr is widespread across archaeal phyla (Supplementary Table S2) and can be easily identified in 30 bacterial phyla (Supplementary Excel File), although bacterial Lhr is distinguished from archaeal Lhr by the addition of a C-terminal 500–600 amino acids of unknown function that lacks obvious sequence homology to other proteins (Figure 5B). Structural homology searches and modelling using bacterial Lhr C-terminal residues against the PHYRE2 and DALI servers identified a region strongly matching protein folds in the DNA glycosylase enzyme AlkZ that contributes to replication-coupled DNA repair [35], and a smaller region matching tandem winged helix domains of the elongation factor SelB [36] (1.3 Å and 6.9 Å RMSD, respectively). We also noted interesting structural similarities between Lhr proteins and the human putative helicase DDX52, data that is presented in supplementary results (Supplementary Figure S7).

We conclude that our analyses indicate that archaeal Lhr proteins most likely target DNA arising at compromised replication forks, which may include RNA–DNA hybrids present as lagging strand Okazaki fragments. We propose that remodeling of fork DNA after binding by Lhr generates ssDNA for ATP-dependent DNA translocation to unwind the fork as part of DNA repair.

## Abbreviations

AA: acceptor excitation
APBS: all-photon burst search
DCBS: dual-channel burst search
HJ: Holliday junction
Lhr: large helicase-related
MPE: manganese-dependent phosphodiesterase
WHD: winged helix domain
