# Supplementary material for: Mechanistic insights into Lhr helicase function in DNA repair
Source: Biochem J. 2020 Aug 19;477(16):2935–47. doi: 10.1042/BCJ20200379 (PMC7437997; doi:10.1042/BCJ20200379)
Supplement: Supplementary Tables S1-S2 and Figures S1-S7 [file BCJ-477-2935-s1.pdf]

Supplementary Data

Table S1: DNA oligonucleotide sequences shown 5' to 3'. For the FRET fork DNA (fork 2) the coupling positions of Atto532 (green) and Atto647N (red) fluorophore are highlighted.

Name		Sequence and modifications of DNA oligonucleotides (5' to 3')
Linear duplex	1	ATCGATAGTCTCTAGACAGCATGTCCTAGCAAGCCAGAATTCGGCAGCGT
	2	ACGCTGCCGAATTCTGGCTTGCTAGGACATGCTGTCTAGAGACTATCGAT
3'-tailed duplex	1	Strand 1 above
	2	GGACATGCTGTCTAGAGACTATCGAT
5'-tailed duplex	1	Strand 1 above
	2	ACGCTGCCGAATTCTGGCTTGCTAGG
Gapped 70mer duplex	1	GCAGGATCCGTATCCGTAACTGGAGCTCTTCGAAGGCCATCGTCGCGAACG ATCCTGCCTAGGGAGCTCC
	2	GGAGCTCCCTAGGCAGGATCG
	3	CGAAGAGCTCCAGTTACGGATACGGATCCTGC
**HJ1** (aka J12)	1	Strand 1 in the linear duplex
	2	GACGCTGCCGAATTCTGGCTTGCTAGGACATCTTTGCCCACGTTGACCC
	3	TGGGTCAACGTGGGCAAAGATGTCCTAGCAATGTAATCGTCTATGACGTT
	4	CAACGTCATAGACGATTACATTGCTAGGACATGCTGTCTAGAGACTATCGA
**HJ2** (aka J6)	1	Strand 1 in the linear duplex
	2	GACGCTGCCGAATTCTGGCTTGCTAGGACATTCTTTGCCCACGTTGACCC
	3	GGGTCAACGTGGGCAAAGAATGTCCTACGTCCGATACGGATAATCGCCAT
	4	ATGGCGATTATCCGTATCGGACGTAGGACATGCTGTCTAGAGACTATCGA
**Fork 1**	1	Strand 1 in the linear duplex
	2	Strand 2 in the linear duplex
	3	GACGCTGCCGAATTCTGGCTTGCTAGGACATCTTTGCCCACGTTGACCC
	4	TGGGTCAACGTGGGCAAAGATGTCC
**Fork 2**	1	Strand 1 in the linear duplex
	2	Strand 2 in the linear duplex
	3	GACGCTGCCGAATTCTGGCTTGCTATGTAACTCTTTGCCCACGTTGACCC
	4	GGGTCAACGTGGGCAAAGAGTTACA
**Fork 3**	1	Strand 1 in the linear duplex
	2	Strand 3 in Fork 2
Fork2 FRET	1	Biotin- ATCGATAGTCTCTAGACAGTATGTCCTAGCAAGCCAGAATTCGGCAGCGT
	2	GGACATACTGTCTAGAGACTATCGAT
	3	GACGCTGCCGAATTCTGGCTTGCTATGTAAATCTTTGCCCACGTTGACCC
	4	GGGTCAACGTGGGCAAAGATTTACA

Table S2: Summary of the phylogenetic distribution of Lhr in archaea. The nomenclature given for archaeal phyla was composed from the Genome Taxonomy Database (GTD) [1], CVTree [2] and the NCBI Taxonomy Browser. These were used as organism identifiers in searches against the non-redundant protein sequences database in NCBI Blastp for homologues of the *Methanothermobacter thermautotrophicus* Lhr amino acid query sequence. Lhr was deemed to be present (shown green) if it contained at least one match in each sub-group/class with an expected value (E) of 0.0, or if the E value of the top sequence match was greater than 0.0 but in pairwise alignment with Mth Lhr using ClustalW we identified conservation of two amino acid residues required for helicase activity in bacterial "core" Lhr (Arg-291 and W575 using the amino acid numbering of Mth Lhr) [3] and which are not part of the canonical helicase/ATPase motifs that are widespread in nature.

**Phylum/grouping**	**Lhr?**	**Additional information**
Aenigmarchaeota – DPANN superphylum		
Aigarchaeota		
Atlaarchaeota		
Asgardarchaeota		Including in C*andidatus* Prometheoarchaeum syntrophicum strain MK-D1 [4] and throughout all of Loki-, Thor-, Odin-, and Heimdallarchaeota.
Crenarchaeota – TACK superphylum		
Euryarchaeota		*Methanothermobacter thermautotrophicus.*
Hadarchaeota		GTD – phylum is represented by two assembled sequences.
Halobacterota		
Huberarchaeota – DPANN		
Hydrothermarchaeota		
Iainarchaeota – DPANN		GTD – the 14 families comprising this phylum were not found in NCBI protein BLAST.
Korarchaeota – TACK		
Macrochaeta		
Nanoarchaeota		Including *Nanoarchaeum equitans*
Parvarchaeota – DPANN		
Thaumarchaeota – TACK		
EX4484-52		GTD – phylum is represented by a single sequence.

Figure S1. Coomassie stained SDS-PAGE acrylamide gel showing 1 μg of purified Lhr used in this work.

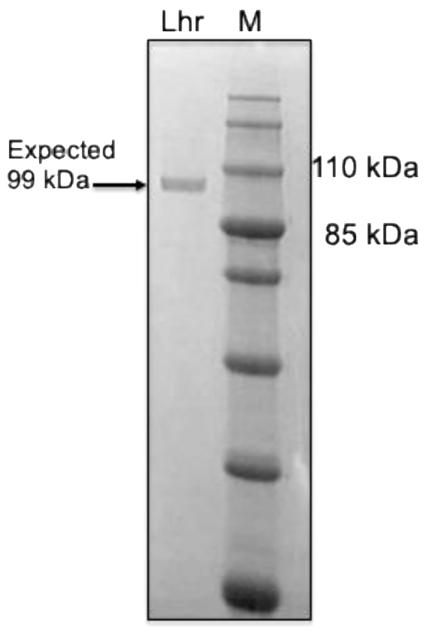

Figure S2. Summary of Lhr unwinding gapped duplex DNA (Figure 2) in reaction buffer containing increasing concentration of ATP (x-axis) and varying concentrations of magnesium chloride: 1 mM (black), 2 mM (blue), 3 mM (brown) and 5 mM (gold). Reactions were in triplicate and standard error is shown. It established that a 1:2 mM magnesium ratio gave optimal unwinding of this substrate.

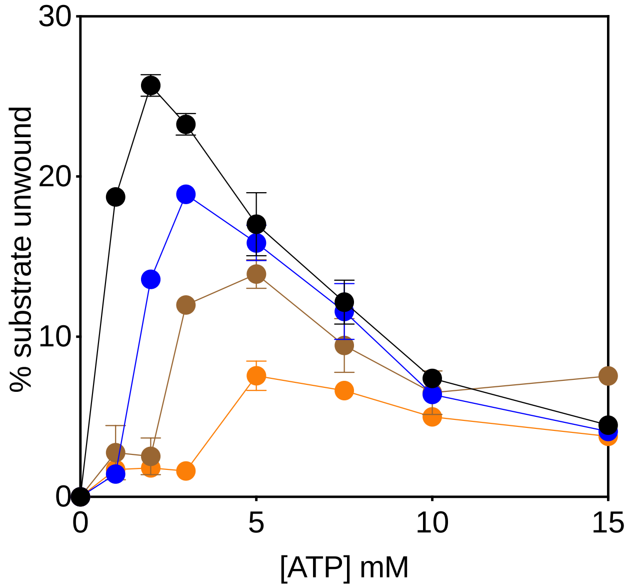

Figure S3. Summary of Lhr (0, 5, 10, 20, 40, 80, 160 and 320nM) unwinding a flayed duplex (20 nM) comprised of DNA or an RNA-DNA hybrid as indicated above the panel. ‘B’ indicates a boiled reaction. Samples were electrophoresed through a 10% acrylamide TBE gel.

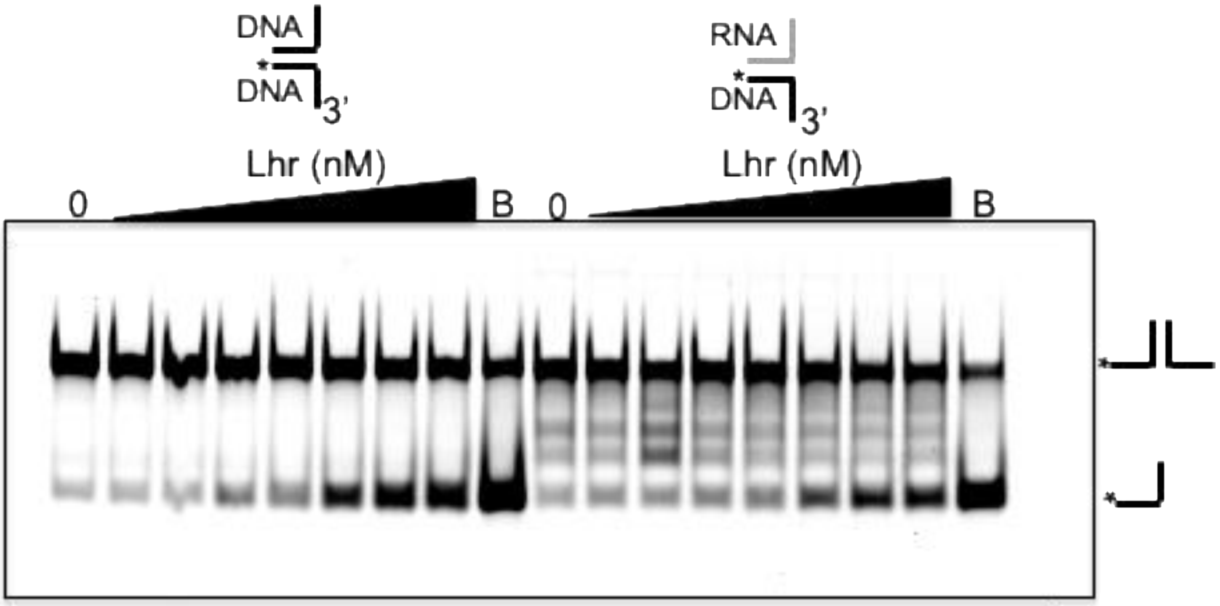

Figure S4. Comparison of Holliday junction (HJ) dissolution products of Lhr and RuvAB. In all panels the asterisk indicates the position of the ^{32}P DNA 5'-end label. **A.** Shows two gel panels; RuvAB (40 nM) catalysed unwinding of Holliday junction (1 nM) into a single flayed duplex product only, alongside a no protein control (0) in 1 mM $MgCl_2$ and 2 mM ATP; products from the same assay of RuvAB, alongside products from titration of Lhr (0, 5, 10, 50 nM) mixed with HJ1 under the same assay conditions. **B.** Shows the same gel as in Figure 2D inset extended to show adjacent lanes containing ssDNA products from Lhr unwinding the 3' tailed duplex DNA.

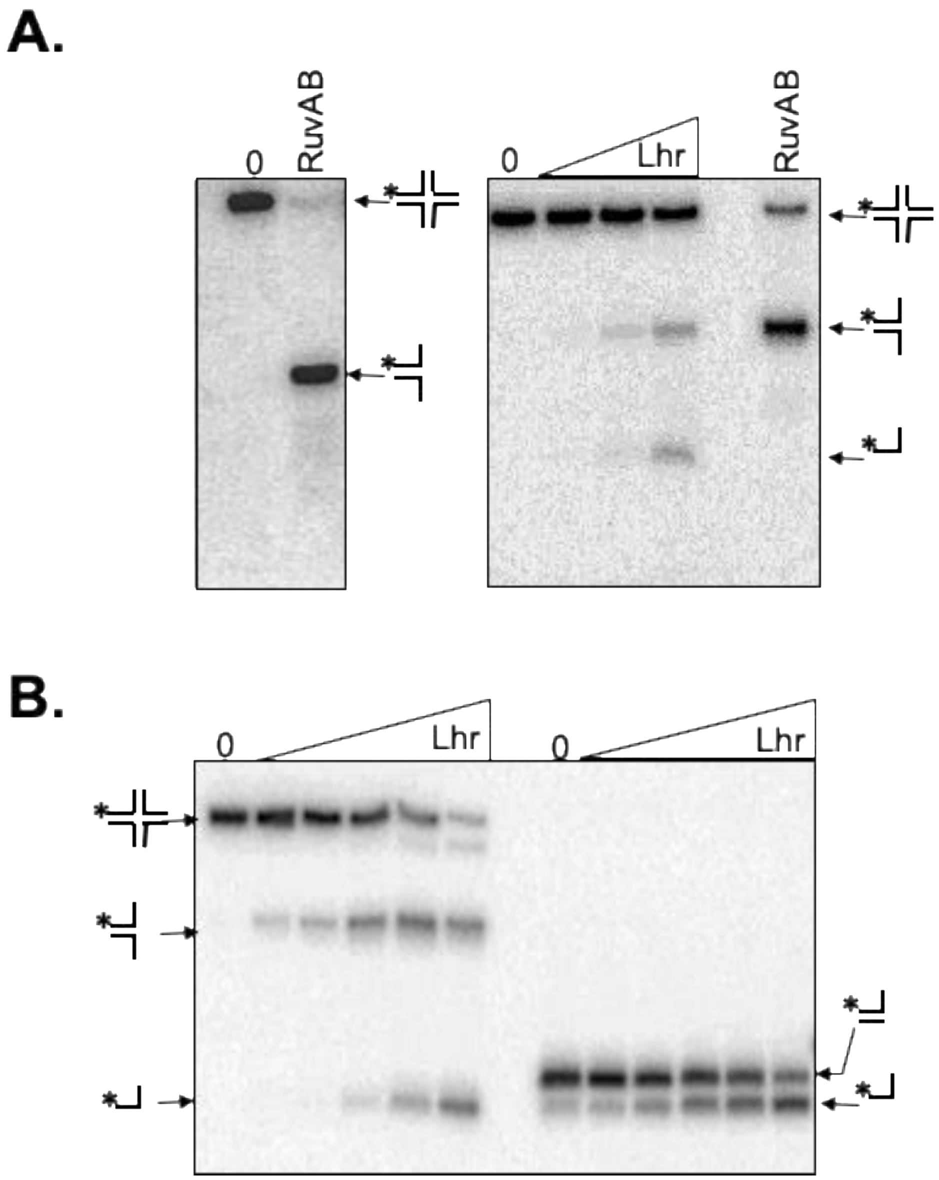

Figure S5. Summary of products formed in 30-minute end-point assays from ATP-dependent unwinding of forked and Holiday junction DNA by Lhr (100 nM).

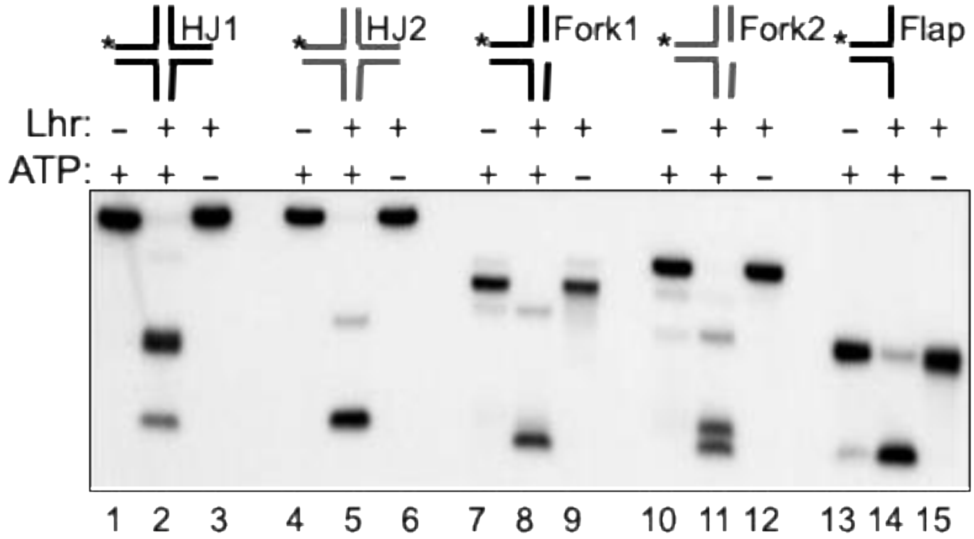

Figure S6. Mean FRET efficiencies and standard deviations for smFRET measurement of Lhr and donor/acceptor labelled fork 2. The mean FRET efficiencies (black line) and standard deviation (grey area) of three technical replicates are shown.

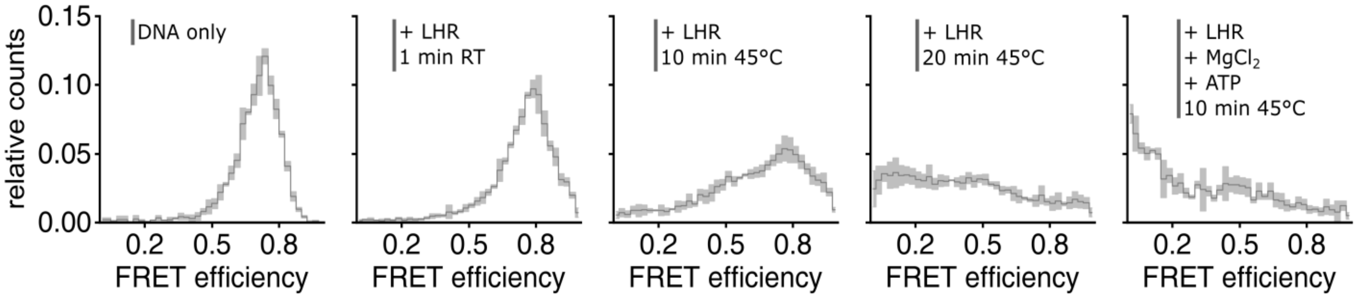

Additional notes: FRET efficiencies equal distances of	
E	Distance (nm)
0.01	>12
0.12	8.22
0.50	5.90
0.72	5.04
0.78	4.78
0.92	3.93

Supplementary Data and Figure S7. Structural comparison of Lhr with human DDX52.

The DDX52 helicase model superimposes less optimally to Lhr (RMSD 2.9Å) but the core domain juxtaposition is apparent to the tandem RecA and winged helix domains. Although there is no equivalent large C-terminal domain as with Lhr (figure S7), small additional DDX52 N- and C-terminal extensions are apparent in the model, located close to the 3'- end of the ssDNA in the Lhr structure. The N-terminal extension partly comprises a 4-helical bundle, identified using the N-terminal PWI region of BRR2 helicase [5] as a Phyre2 modelling template (figure S7 (B) and inset). This 4-helical bundle exhibits structural similarity to other nucleic acid processing enzymes, including the fingers sub-domain of family A DNA polymerases [6] and REC2 domain of Cas9 nucleases [7], potentially indicating a role for the 4-helical bundle in strand displacement or DNA binding, respectively. This indicates a potential role for appended domains onto core Lhr-type helicase folds in nucleic acid manipulation during action of the core helicase.

Structural model of DDX52 superimposed onto *M. smegmatis* Lhr (PDB: 5V9X), with Lhr shading as in (A), and DDX52 model shaded in light blue. Inset: DDX52 N-terminal 4 helical bundle (aa 1-64, light blue) superimposed onto *E. coli* DNA polymerase I (green, PDB: 1D8Y [6], RMSD 4.5Å), *Neisseria meningitidis* Cas9 nuclease (orange, PDB: 6JDQ, RMSD 3.9Å) and *Saccharomyces cerevisiae* BRR2 helicase (red, PDB: 5DCA, RMSD 0.8Å).

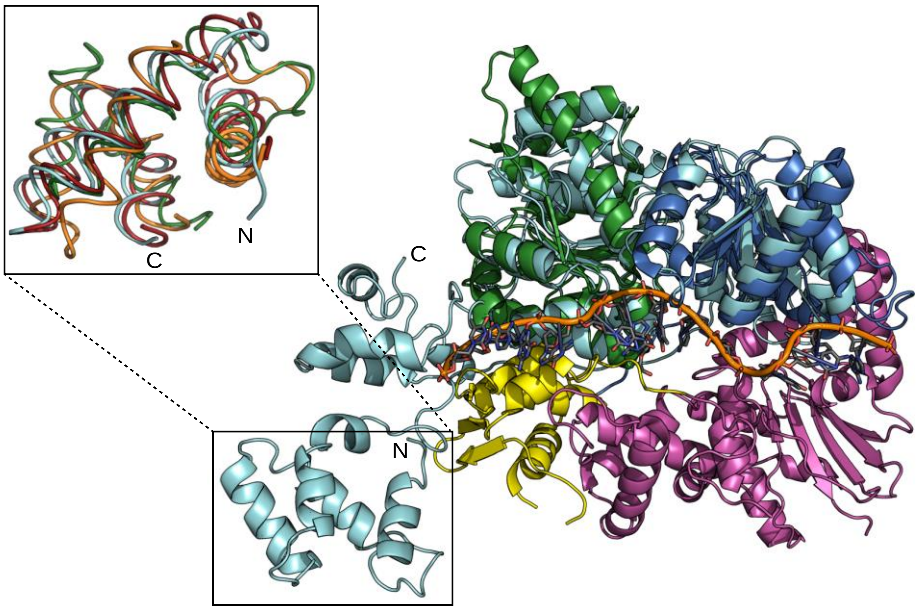

Supplementary References

1 Chaumeil, P. A., Mussig, A. J., Hugenholtz, P. and Parks, D. H. (2019) GTDB-Tk: a toolkit to classify genomes with the Genome Taxonomy Database. Bioinformatics

2 Qi, J., Wang, B., and Hao, B. (2004) Whole genome prokaryote phylogeny without sequence alignment: A K-string composition approach. . J. Mol. Evol. **58**, 1-11

3 Ejaz, A., Ordonez, H., Jacewicz, A., Ferrao, R. and Shuman, S. (2018) Structure of mycobacterial 3'-to-5' RNA:DNA helicase Lhr bound to a ssDNA tracking strand highlights distinctive features of a novel family of bacterial helicases. Nucleic Acids Res. **46**, 442-455

4 Imachi, H., Nobu, M. K., Nakahara, N., Morono, Y., Ogawara, M., Takaki, Y., Takano, Y., Uematsu, K., Ikuta, T., Ito, M., Matsui, Y., Miyazaki, M., Murata, K., Saito, Y., Sakai, S., Song, C., Tasumi, E., Yamanaka, Y., Yamaguchi, T., Kamagata, Y., Tamaki, H. and Takai, K. (2020) Isolation of an archaeon at the prokaryote-eukaryote interface. Nature. **577**, 519-525

5 Absmeier, E., Rosenberger, L., Apelt, L., Becke, C., Santos, K. F., Stelzl, U. and Wahl, M. C. (2015) A noncanonical PWI domain in the N-terminal helicase-associated region of the spliceosomal Brr2 protein. Acta Crystallogr D Biol Crystallogr. **71**, 762-771

6 Teplova, M., Wallace, S. T., Tereshko, V., Minasov, G., Symons, A. M., Cook, P. D., Manoharan, M. and Egli, M. (1999) Structural origins of the exonuclease resistance of a zwitterionic RNA. Proc Natl Acad Sci U S A. **96**, 14240-14245

7 Sun, W., Yang, J., Cheng, Z., Amrani, N., Liu, C., Wang, K., Ibraheim, R., Edraki, A., Huang, X., Wang, M., Wang, J., Liu, L., Sheng, G., Yang, Y., Lou, J., Sontheimer, E. J. and Wang, Y. (2019) Structures of Neisseria meningitidis Cas9 Complexes in Catalytically Poised and Anti-CRISPR-Inhibited States. Mol Cell. **76**, 938-952 e935
